# Immunodominant PstS1 antigen of *mycobacterium tuberculosis *is a potent biological response modifier for the treatment of bladder cancer

**DOI:** 10.1186/1471-2407-4-86

**Published:** 2004-11-26

**Authors:** Christian Sänger, Andreas Busche, Gabriele Bentien, Ralf Spallek, Fatima Jonas, Andreas Böhle, Mahavir Singh, Sven Brandau

**Affiliations:** 1 Division of Immunotherapy, Research Center Borstel, Parkallee 26b, 23845 Borstel, Germany; 2 LIONEX Diagnostics & Therapeutics GmbH, Mascheroder Weg 1 b, 38124 Braunschweig, Germany; 3 Helios Agnes Karll Hospital, Lübecker Str. 18–20, 23611 Bad Schwartau, Germany

## Abstract

**Background:**

Bacillus Calmette Guérin (BCG)-immunotherapy has a well-documented and successful clinical history in the treatment of bladder cancer. However, regularly observed side effects, a certain degree of nonresponders and restriction to superficial cancers remain a major obstacle. Therefore, alternative treatment strategies are intensively being explored.

We report a novel approach of using a well defined immunostimulatory component of *Mycobacterium tuberculosis *for the treatment of bladder cancer. The phosphate transport protein PstS1 which represents the phosphate binding component of a mycobacterial phosphate uptake system is known to be a potent immunostimulatory antigen of *M. tuberculosis*. This preclinical study was designed to test the potential of recombinant PstS1 to serve as a non-viable and defined immunotherapeutic agent for intravesical bladder cancer therapy.

**Methods:**

Mononuclear cells (PBMCs) were isolated from human peripheral blood and stimulated with PstS1 for seven days. The activation of PBMCs was determined by chromium release assay, IFN-γ ELISA and measurement of lymphocyte proliferation. The potential of PstS1 to activate monocyte-derived human dendritic cells (DC) was determined by flow cytometric analysis of the marker molecules CD83 and CD86 as well as the release of the cytokines TNF-α and IL-12. Survival of presensitized and intravesically treated, tumor-bearing mice was analyzed by Kaplan-Meier curve and log rank test. Local and systemic immune response in PstS1-immunotherapy was investigated by anti-PstS1-specific ELISA, splenocyte proliferation assay and immunohistochemistry.

**Results:**

Our *in vitro *experiments showed that PstS1 is able to stimulate cytotoxicity, IFN-γ release and proliferation of PBMCs. Further investigations showed the potential of PstS1 to activate monocyte-derived human dendritic cells (DC). *In vivo *studies in an orthotopic murine bladder cancer model demonstrated the therapeutic potential of intravesically applied PstS1. Immunohistochemical analysis and splenocyte restimulation assay revealed that local and systemic immune responses were triggered by intravesical PstS1-immunotherapy.

**Conclusion:**

Our results demonstrate profound *in vitro *activation of human immune cells by recombinant PstS1. In addition, intravesical PstS1 immunotherapy induced strong local and systemic immune responses together with substantial anti-tumor activity in a preclinical mouse model. Thus, we have identified recombinant PstS1 antigen as a potent immunotherapeutic drug for cancer therapy.

## Background

Urothelial carcinoma of the bladder accounts for about 4% of all cancer related death in man. The large majority of tumors (70–80%) is superficial at diagnosis and has a high rate of local recurrence (70%) and progression (30%) after local surgical therapy. Therefore, patients require lifelong medical follow-up examinations and effective prophylactic treatment to prevent recurrences and progression of the tumor.

In this type of cancer, the immunotherapeutic use of Mycobacteria – specifically Bacillus Calmette-Guerin (BCG), a non-pathogenic strain of *Mycobacterium bovis *– has a well-documented and successful clinical history. Immunotherapy with BCG is performed by six weekly instillations of viable Mycobacteria (for induction course) into the bladder of patients after initial transurethral resection of the tumor. Until now various clinical trials have shown that this type of therapy is superior to topical chemotherapy and transurethral resection of the tumor alone to prevent recurrences and local progression especially in patients with high risk tumors [1-3]. Despite several clear advantages of BCG immunotherapy for the treatment of bladder cancer, several problems and limitations compromise its use. Although BCG is the most effective agent against superficial transitional cell carcinoma (TCC), currently there are still 30 to 40% of patients not responding to the therapy [4]. Furthermore, in the case of muscle invasive bladder cancer, BCG has not been shown to be effective [5]. BCG's activity appears to be strictly localized and as a living organism, BCG poses unique toxicity problems associated with its use. Although only 5% of these problems are severe, most if not all patients experience some irritable bladder symptoms (cystitis) during BCG therapy [6]. Roughly 40% develop hematuria and 30% experience flu-like symptoms including fever, malaise and nausea or vomiting. Actual BCG sepsis is a rare event and has been reported in only 0.4% of all cases. In addition, some of these cases have been fatal [7,8]. Thus, the reported side effects limit the clinical applicability and acceptance of this effective immunotherapy and underscore the need for alternative forms of treatment.

In order to limit toxicity recent endeavors are therefore focused on the development of alternative non-viable products and some of those have already been tested in preclinical and clinical studies [9-12]. The mycobacterial antigen PstS1 is known as a highly immunogenic and immunostimulatory component of the mycobacterial cell membrane [13]. PstS1 is the phosphate binding subunit of the inorganic phosphate uptake system from *M. tuberculosis *belonging to the family of ABC (ATP-binding cassette) transporters [14,15]. It is a glycosylated lipoprotein which can be found both, intracellularly and secreted into the extracellular culture supernatant [16,17]. Moreover PstS1 represents one of the most immunogenic antigens in active multibacillary tuberculosis [18].

We hypothesized that this highly immunogenic protein antigen could function as an effective biological response modifier in immunotherapy of bladder cancer. To test the tumortherapeutic potential of recombinantly expressed PstS1 [19] we performed a detailed analysis of the immunostimulatory capacity of this antigen in a well-defined human *in vitro *system and in a previously described murine model of experimental bladder cancer therapy [20]. Because the role of prior exposure of bladder cancer patients to mycobacterial antigens for the effectiveness of BCG therapy is controversely discussed [21-23], we performed intravesical PstS1-immunotherapy with and without prior sensitization of mice.

The data reported herein demonstrate that PstS1 is a potent activator of human tumor-cytotoxic MNCs, induces maturation and activation of human dendritic cells and most importantly is very effective in the treatment of experimental orthotopic bladder cancer. While local and systemic immune responses were observed in sensitized and non-sensitized mice, immunotherapy of cancer was only successful in non-sensitized animals.

## Methods

### Cell culture

The human bladder tumor cell line T-24 was cultured at 37°C and 5% CO_2 _in RPMI 1640 (PAA Laboratories, Linz/Austria) containing 10% FCS (Linaris, Bettingen/Germany), 1% glutamine, 100 U/ml penicillin and 100 μg/ml streptomycin. The murine bladder tumor cell line MB-49 was cultured at 37°C and 5% CO_2 _in DMEM (PAA Laboratories, Linz/Austria) containing 10% FCS (Linaris, Bettingen/Germany), 1% L-glutamine, 100 U/ml penicillin and 100 μg/ml streptomycin.

### Stimulation of human PBMCs

MNCs from heparinized blood of healthy human donors were obtained by discontinuous gradient centrifugation using Biocoll Separating Solution (Biochrom, Berlin/Germany) and adjusted to a concentration of 1 × 10^6^/ml in RPMI-1640 medium containing 5% human serum, 100 U/ml penicillin and 100 μg/ml streptomycin. Recombinant PstS1 in concentrations from 0.1 μg/ml – 100 μg/ml, BCG (Connaught substrain, Immucyst, 4 × 10^4 ^cfu/ml), or PBS (100 μl) was added and the cells were cultured for 7 days in 6-well microtiter plates at 37°C and 5% CO_2_.

### Chromium release assay

Cytotoxicity was determined in a standard 4-hour chromium release assay. Target cells were labeled with Na_2_^51^CrO_4 _(ICN, Irvine/USA) for 1 h at 37°C, washed and resuspended at 5 × 10^4 ^cells/ml. Effector cells were added to a total of 100 μl of target cells at an effector:target ratio of 40:1. The radioactive content of the supernatant was measured in a gamma-counter (Berthold, Wildbad/Germany). The specific lysis was determined according to the formula: spec. lysis (%) = 100 × (Exp – Spo)/ (Max – Spo) where Exp is the experimental release, Spo the spontaneous release and Max the maximum release.

### Human IFN-γ ELISA

Supernatants of PstS1, BCG and PBS stimulated PBMCs were recovered at days 2, 5 or 7 and examined for the presence of IFN-γ. Each experiment was carried out several times with different donors. Detection was performed with an anti human IFN-γ ELISA-Kit according to the manufacturers' instructions (eBioscience, San Diego/USA).

### Proliferation of human PBMC

PBMCs were stimulated for 2 to 7 days with PstS1, BCG, PBS or PHA (Sigma). 2 × 10^5 ^cells / well of the stimulated PBMCs were cultured in a microwell plate and 1 μCi ^3^H-Thymidine (five wells per sample) (Amersham, Freiburg/Germany) was added during the last 18 h of stimulation. DNA was harvested on filter membranes and thymidine incorporation was measured by liquid scintillation counting (counter: LKB Wallace 1205 beta-plate, Turku/Finland).

### Generation and stimulation of human monocyte derived dendritic cells

After separating peripheral blood mononuclear cells (PBMCs) from heparinized blood of healthy donors by Ficoll-Paque centrifugation, monocytes were elutriated by counterflow centrifugation. For generation of immature DCs, 2 × 10^6 ^monocytes were cultured for seven days with 2 ml RPMI 1640, 10% FCS (Linaris, Bettingen/Germany) 1% penicillin/streptomycin and IL-4/GM-CSF (500 U/ml each) (TEBU/PeproTech, Offenbach/Germany) in 24-well cell culture plates (Nunc, Wiesbaden/Germany). Exchange of medium was carried out at day three and day five. This procedure resulted in full differentiation of monocytes with no undifferentiated monocytes present in the cultures after seven days. Immature DCs were stimulated for three days with 10 μg/ml PstS1 or BCG (MOI = 0.01) and subjected to flow cytometry. Debris was excluded from the analysis according to FSC/SSC gating.

### Flow cytometry

Expression of cell surface molecules was analyzed by flow cytometry (FACSCalibur, Becton Dickinson, Franklin Lakes/USA) using phycoerythrin (PE) conjugated monoclonal antibodies (mAbs): anti-CD1a (Biosource, Camarillo/USA), anti-CD83 (Pharmingen, San Diego/USA), anti-CD14 and anti-CD86 (Dianova, Hamburg/Germany).

1 × 10^5 ^DCs suspended in PBS containing 5% human serum and 0.1 % sodium acide were incubated with mAbs for 30 minutes on ice. Then cells were washed with PBS and resuspended in 400 μl 1.5 % paraformaldehyde in PBS.

### human TNF-α and IL-12 ELISA

Supernatants of PstS1, BCG and PBS stimulated DCs were recovered at day 3 after stimulation and examined for the presence of TNF-α and IL-12. Each experiment was carried out several times with different donors. TNF-α detection was performed with a quantitative ELISA, provided by Dr. H. Gallati (Intex, Muttenz/Switzerland).

IL-12 detection was carried out with an ELISA-Kit from eBioscience (San Diego/USA).

### PLG-Particle preparation and protein loading

PLG-particles (10 mg/ml) (Lionex, Braunschweig/Germany) were diluted 4:1 with PBS and the pH was adjusted to 7.0 with NaOH. PstS1 was recombinantly expressed in *E. coli *and purified by standard chromatography.

250 μg of PLG-particles were loaded with PstS1 or BSA by coincubation with 60 μg of the respective protein for 15 h at RT on a shaker (overall volume 100 μl). Afterwards the particles were spun down and washed for 5 min with 100 μl PBS. The supernatant was removed and the particles were diluted in 100 μl of PBS. 10 μl of loaded particle solution were centrifuged and the pellet was treated for 30 min at RT with 0.1 M NaOH/ 10% SDS. After centrifugation in a minifuge, supernatant was removed and analyzed on a 10% SDS page. Using recombinant PstS1 as a calibration standard it was determined that 250 μg PLG-particles bound approximately 50 μg of PstS1 protein.

### PLG-particle vaccination and immunotherapy of experimental bladder cancer

Female C57BL/6 mice were purchased from Charles-River Laboratories (Sülzfeld/ Germany) at the age of 6–8 weeks. To test efficacy of PstS1 therapy in C57/BL6 mice a published syngeneic, orthotopic bladder cancer model was used [10]. Trial I: 15 animals per group were subcutaneously injected with 250 μg PLG-particles coated with 50 μg PstS1 or 250μg PLG-particles alone. Ten days later the mice were anesthetized by intraperitoneal treatment with Pentobarbital (0.067 mg/g body weight). After insertion of a 24-gauge teflon intravenous catheter (Insyte-W, Becton Dickinson, Franklin Lakes/USA) transurethrally into the bladder, electrocoagulation was performed with a guide wire. Thereafter 6 × 10^4 ^MB-49 cells were instilled into the bladder. Intravesical immunotherapy with 100 μg PstS1 in 100 μl PBS was performed on days 1, 8, 15 and 22 after tumor implantation. Control groups were instilled with PBS (100 μl) alone. The viability status of the mice was checked daily. Surviving mice were sacrificed on day 70. Survival of mice was compared using Kaplan-Meier analysis and log-rank test. Trial II: Performance similar to trial I with the following differences. 11 animals per group were subcutaneously injected with a) 250 μg empty PLG-particles b) 250 μg PLG-particles coated with 50 μg BSA c) 100μl of PBS. Intravesical treatments were performed with 100 μg PstS1 or PBS only.

### Immunohistochemistry of murine bladders

C57/BL6 mice (5 per group) were treated as described in table I. One day after the fourth instillation animals were sacrificed. Bladders were dissected immediately, shock-frozen in liquid nitrogen and stored at -80°C. The immunohistochemistry and analysis of cellular influx of different leukocyte subsets was performed on 5μm frozen sections as described previously [24].

### anti-PstS1-IgG ELISA from murine blood

Murine blood was obtained immediately after sacrificing mice by heart punctation and centrifuged in a microcentrifuge to obtain the serum for antibody analysis which was performed with the PstS1-ELISA-kit (Lionex GmbH, Braunschweig/Germany) according to the manufacturer's instructions.

### Splenocyte restimulation assay

Splenocytes were isolated, washed with PBS and erythrocytes were lysed with H_2_O. Afterwards 2 × 10^5 ^cells / well were cultured in a microwell plate and stimulated with 10 μg/ml PstS1 for a period of five days (five wells per sample). Then 1μCi ^3^H-Thymidine (Amersham, Freiburg/Germany) was added and cells were incubated for 15 h at 37°C and 5% CO_2_. Finally the DNA was harvested on filter membranes and ^3^H-Thymidine incorporation was measured by liquid scintillation counting (counter: LKB Wallace 1205 beta-plate, Turku/Finland).

## Results

### PstS1 activates human PBMCs

To assess the immunostimulatory properties of our PstS1-preparation we analyzed several human PBMC *in vitro *systems. As in these systems the immunostimulatory mechanisms of whole BCG bacteria have been thoroughly studied in the past [9,25-27] we used BCG as a positive control and reference stimulus.

**Figure 1:**
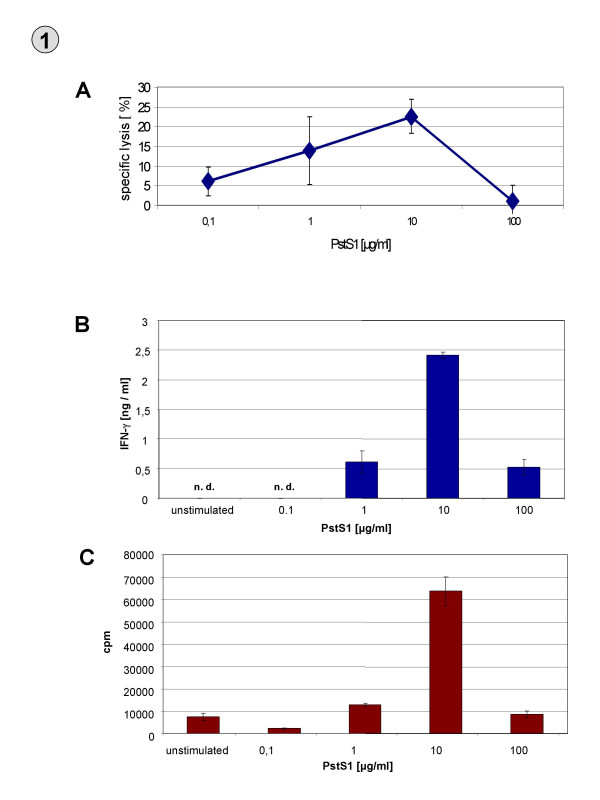
**Activation of PBMC by PstS1 is dose-dependent**. PBMCs were stimulated for 7 days with different PstS1 concentrations in a range from 0.1 μg/ml to 100 μg/ml. **A **4-hour chromium release assay against T-24 bladder tumor cells. Effector-target cell ratio of 40:1 **B **IFN-γ release was determined by ELISA from supernatants of the stimulated PBMCs **C **PBMC proliferation was measured by overnight ^3^H-Thymidine incorporation assay (1 μCi/2 × 10^5 ^cells). One representative experiment out of 3–6 is shown (mean ± SD). n.d. = not detectable

In a first set of experiments the optimal concentration of PstS1 to stimulate human PBMC cytotoxicity (Fig. 1A), IFN-γ release (Fig. 1B) and proliferation (Fig. 1C) was determined. Using a concentration range from 0.1 μg/ml to 100 μg/ml PstS1 showed a typical bell-shaped dose response curve. A concentration of 10 μg/ml was found to be optimal for stimulation of human PBMC in all three readout systems. In order to analyze the time course of human PBMC activation, a time kinetic study of PBMC stimulation was performed. BCG, which has been previously described to induce potent anti-tumor cytotoxicity in human PBMCs [27] was used as a reference stimulus and positive control.

**Figure 2:**
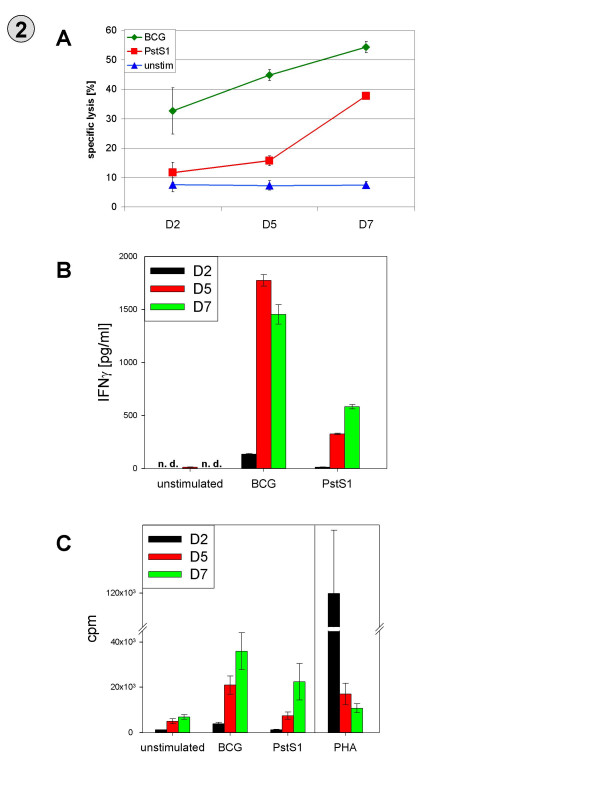
**Time-course of PBMC stimulation**. PBMC were stimulated with PstS1 (10μg/ml), BCG (4 × 10^4 ^CFU/ml), PHA (2,5 μg/ml) or left unstimulated. Cytotoxicity (A), IFN-γ-production (B) and proliferation (C) of PBMC were measured after 2, 5 and 7 days of stimulation. **A **Cytotoxicity assay. 4-hour chromium release assay against T-24 bladder tumor cells. Effector-target cell ratio of 40:1. **B **IFN-γ release measured by ELISA from culture media of PBMC. **C **PBMC proliferation measured by overnight ^3^H-Thymidine incorporation assay (1 μCi/2 × 10^5 ^cells). For A and B one representative experiment out of 3 is shown (mean ± SD), for C a composite of seven independent experiments is shown (mean ± SEM). For PHA-stimulation one out of two experiments is shown (mean ± SD) . n.d. = not detectable

As depicted in figure 2A,2B,2C activation of human PBMC by PstS1 was time-dependent with the strongest activation on day 7 and only marginal activation at early time points (day 2). Overall, in this study we have tested the activation of PBMC of ten different human donors in response to PStS1 stimulation. As expected, we observed a certain degree of donor variability in this assays with e.g. IFN-γ induction ranging from 500 pg/ml to 5 ng/ml. Comparison with BCG consistently indicates a similar time-kinetic of PBMC activation between these two biological response modifiers albeit with higher absolute levels of activation induced by BCG. This kinetic of activation is contrasted by mitogenic stimulation with PHA (figure 2c) or ConA (not shown) which show an expected peak of PBMC stimulation at early time points with a subsequent dramatic decrease.

### Dendritic cell activation by PstS1

Dendritic cells are central cellular mediators for the induction of anti-tumor immune responses. We tested the potential of PstS1 to induce activation and maturation of human monocyte-derived DCs.

**Figure 3:**
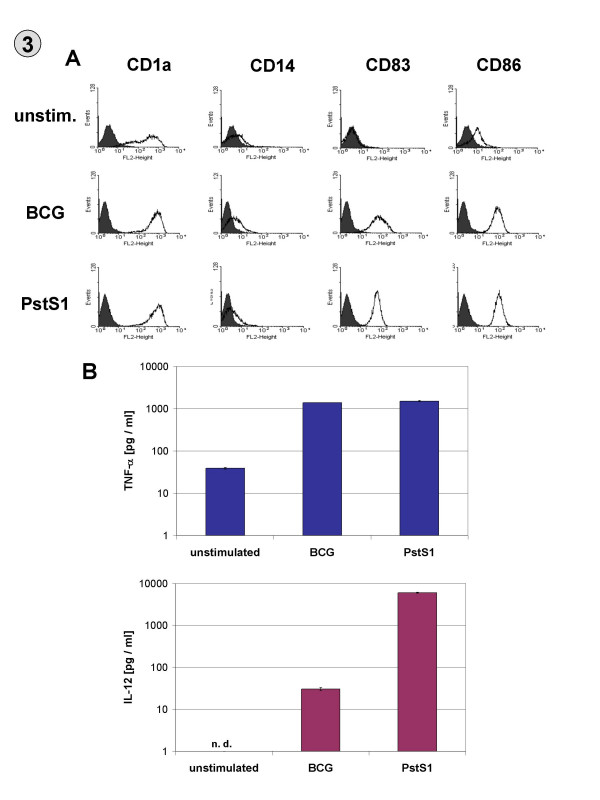
**Activation of human monocyte-derived dendritic cells by PstS1**. **A **Monocytes were differentiated for seven days with GM-CSF and IL-4 (500 U / ml each), stimulated with BCG (MOI = 0.01) or PstS1 (10 μg / ml) for three days and afterwards analyzed by flow cytometry for the expression of CD1a, CD14, CD83 and CD86. Isotype control = filled histograms, specific antibody = open histograms. Cells were gated on intact dendritic cells according to forward scatter and sideward scatter. **B **Supernatants of the stimulated DCs were collected and ELISAs for TNF-α and IL-12p70 were performed. One representative experiment out of 5 is shown (mean ± SD). n.d. = not detectable.

After 7 days of differentiation human monocyte-derived dendritic cells showed the typical immature phenotype with low or absent expression of the monocyte marker CD14 and the maturation markers CD83 and CD86 (Fig. 3A). At the same time immature DCs expressed high amounts of CD1a. Stimulation of dendritic cells with either PstS1 or BCG induced strong upregulation of CD83 and CD86 indicating phenotypical maturation of DC after challenge with PstS1. In parallel, the cytokine response of DC was assessed and PstS1 was found to induce substantial amounts of TNF-α and IL-12 p70, two key cytokines in dendritic cell biology. Interestingly, PstS1 reproduceably induces IL-12p70 in the ng range, while BCG only induced relatively low levels of IL-12p70 (50–500 pg/ml) (Fig. 3B).

### Intravesical immunotherapy with PstS1 in sensitized and non-sensitized mice

After we have shown profound immunostimulatory properties of PstS1 *in vitro *we conducted a series of studies to test its immunotherapeutic potential *in vivo*. Prompted by the controversy about the role of prior exposure to mycobacterial antigens in BCG immunotherapy we wanted to assess the *in vivo *activity of PstS1 in sensitized and non-sensitized mice. For this purpose two independent *in vivo *experiments were carried out. PLG-particles have previously been described as useful agents for sensitization of mice to mycobacterial antigens and were used as such in our study [13].

**Figure 4:**
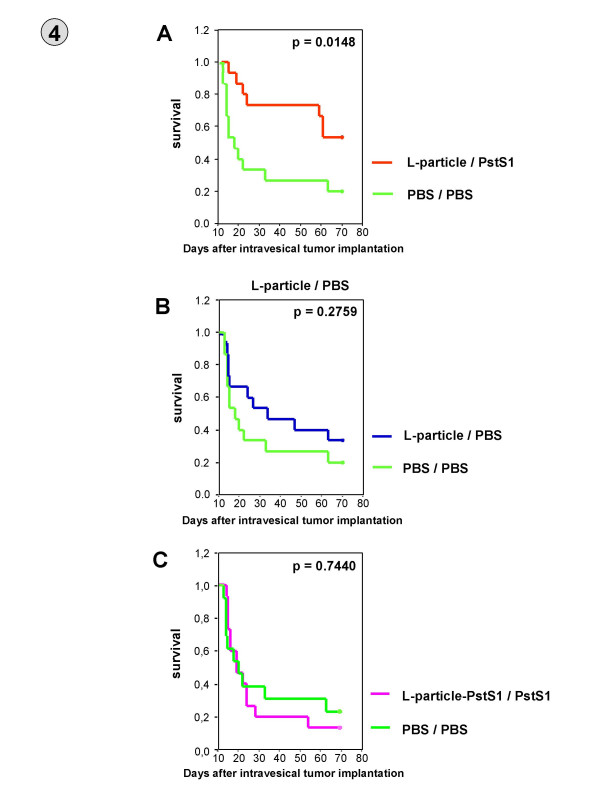
**Intravesical PstS1 immunotherapy in nonsensitized and PstS1-sensitized mice**. Ten days before inoculation of MB-49 tumor cells mice were s.c. sensitized by injection of empty L-particles (A/B) or L-particle loaded with PstS1 (C). On days 1, 8, 15 and 22 after tumor challenge, mice were treated by intravesical instillation of PstS1 (A/C) or PBS (B). Mice which received s.c. PBS and intravesical PBS served as control. Survival of mice was analyzed by Kaplan-Meier curve and log rank test.

**Figure 5:**
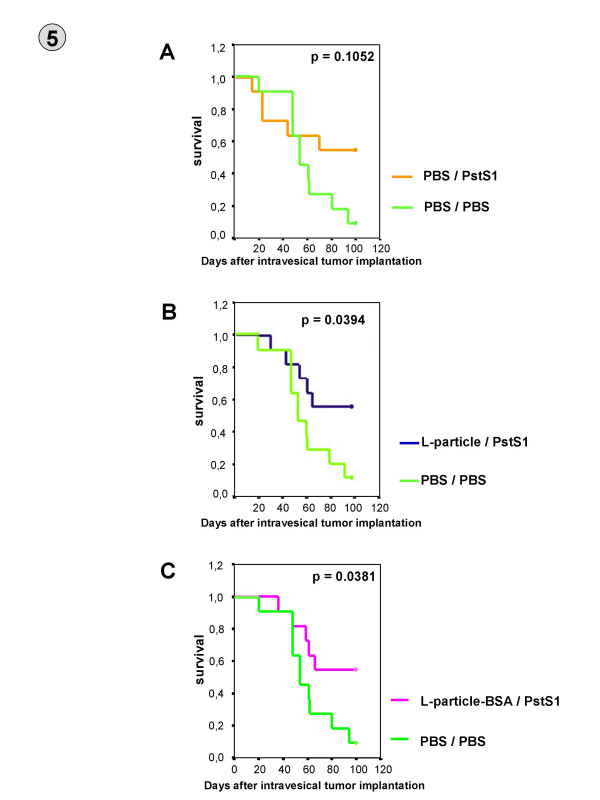
**Intravesical PstS1 treatment after sensitization with PBS, PLG-particles or PLG-particles/BSA**. Ten days before inoculation of MB-49 tumor cells mice were s.c. sensitized by injection of PBS (A), L-particles (B) or L-particles loaded with BSA (C). On days 1, 8, 15 and 22 after tumor challenge, mice were treated by intravesical instillation of PstS1 (A/B/C). Mice which received s.c. PBS and intravesical PBS served as control. Survival of mice was analyzed by Kaplan-Meier curve and log rank test.

In a first series of experiments we analyzed mice which had been s.c. sensitized with empty control particles or with particles loaded with PstS1 antigen. Ten days later mice received inoculation of tumor cells and subsequent intravesical treatment with either PstS1 or PBS control solution (four weekly instillations). Mice which received s.c. PBS followed by intravesical PBS served as negative controls. Using this experimental set up we could show that pre-vaccination with empty particles and intravesical treatment with PstS1 protein significantly prolonged survival of mice and thus provided a clear therapeutic benefit in this model of experimental bladder cancer (Fig. 4a). When mice received s.c. injections of PLG-particles followed by intravesical control PBS, survival of mice was marginally increased suggesting a possible minor non-specific effect of the sensitization procedure (Fig. 4b). Unexpectedly, antigen-specific sensitization with PstS1-loaded particles prior to intravesical PstS1 inoculation completely abrogated the therapeutic effect (Fig. 4c). To further substantiate the findings of trial one a second trial with a modified setup was conducted. In this second trial we compared the effect of s.c. PBS injections, injections of empty PLG-particles and injections of PLG-particles loaded with an irrelevant antigen (BSA) on the effect of intravesical PstS1 treatment. This experimental set up revealed a modest therapeutic benefit of intravesical PstS1 in the absence of prior sensitization (Fig. 5a). The number of mice in this experiment was, however, too small to achieve statistical significance using a stringent log-rank test (p = 0.1052). When intravesical PstS1 instillations were combined with pre-vaccination with empty PLG-particles, the result from the previously described trial was confirmed and again a clearly prolonged survival of mice became evident (Fig. 5b). Based on the hypothesis that the sensitization by itself, irrespective of the antigen used for sensitization, might have impeded the effect of intravesical PstS1 in trial one, we tested the effect of particles loaded with irrelevant BSA protein. However, as shown in Fig. 5c, the therapeutic effect of PstS1 remained virtually unchanged suggesting that the inhibition was not due to the sensitization by itself but rather PstS1 specific.

### Local and systemic immune response in PstS1-immunotherapy

After we had demonstrated successful immunotherapy of bladder cancer by local instillation of PstS1 we next analyzed the local and systemic immune response of the various treatment groups to obtain initial insight into the immunological basis of this novel immunotherapy. To achieve this we analyzed the systemic antibody response by a semiquantitative anti-PstS1 IgG ELISA, the systemic lymphocyte response by splenocyte restimulation assays and the local immune response in the bladder by immunohistology.

**Figure 6:**
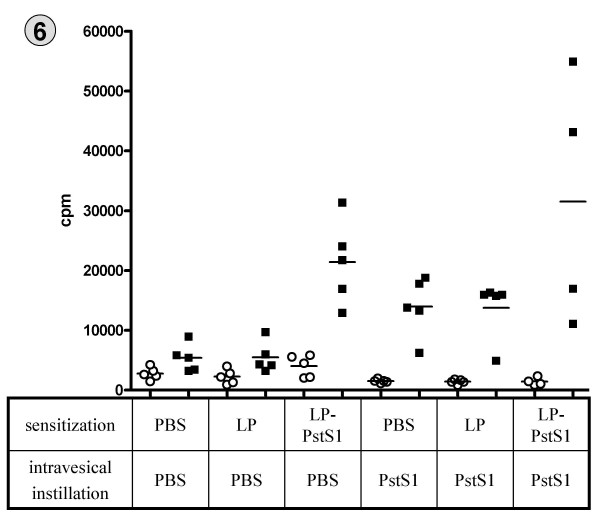
**Splenocyte Restimulation**. Treatment of mice was performed as indicated (s.c. sensitization / intravesical instillation). One day after the fourth intravesical PstS1 treatment splenocytes were isolated and restimulated with PBS as control (open circles) or 10 μg/ml PstS1 (filled boxes) for five days and proliferation was analyzed by ^3^H-Thymidine incorporation assay.

As expected no anti-PstS1 IgG response was detected in the two groups which neither had been sensitized by PstS1 antigen s.c. nor received intravesical inoculations of PstS1. On the other hand, both s.c. and intravesical challenge with PstS1 resulted in positive antibody responses in every animal of the respective groups. This is an interesting finding as it indicates that systemic anti-PstS1 immune responses cannot only be induced by the well established s.c. route but also by intravesical instillations of antigen into the murine bladder (Table 1). Positive antibody responses were also observed in the group which received a combination of s.c. PstS1-loaded particles and intravesical PstS1 and as such failed therapy. A similar picture was observed with regard to the response of restimulated splenocytes (Fig. 6). The two groups of mice with no prior contact to PstS1 only minimally responded to *in vitro *stimulation with PstS1. In contrast, the four groups of mice which had been exposed to PstS1 either s.c. or intravesically or via both routes strongly responded to specific restimulation of their splenocytes. Again these data indicate that systemic immune responses to PstS1 can be achieved by both s.c. and intravesical challenge. While a s.c. sensitization of mice with PstS1 abrogated the anti-tumor effect of intravesical PstS1 (see Fig. 4) the systemic immune response was not negatively affected but even enhanced in some animals (see Fig. 6).

After the analysis of the systemic immune response to PstS1 we continued our experiments with an immunohistological study of the local immune response. To this end we specifically looked at the influx of lymphocytes, dendritic cells, macrophages and granulocytes (table 2). As expected, bladders of control mice with no PstS1 injections (groups 1–2) were only minimally infiltrated by granulocytes (Gr-1 antigen) or CD11b-positive cells (activation marker for granulocytes and macrophages). On the other hand, after local instillation of PstS1 a distinct influx of granulocytes and macrophages was observed (groups 4–5, table 2). This cellular infiltration of the bladder with granulocytes and macrophages was not substantially altered by additional sensitization of the mice with PstS1-loaded particles (group 6, table 2). In contrast to what is known about classical immunotherapy with BCG [24], challenge of mice with PstS1 only induced a moderate infiltration of the bladder with CD4-positive lymphocytes (groups 4–5, table 2). A slight increase in the number of CD8-positive cells was noted in mice which received s.c. PstS1 sensitization in addition to intravesical PstS1 (group 6, table 2). Dendritic cells (CD11c) were a rare cell population in control mice without PstS1 injections (groups 1–2). A slightly increased number of dendritic cells was noted in the bladders of mice after intravesical instillation of PstS1. Nonetheless, the overall local immune response in the bladder during PstS1 immunotherapy is clearly dominated by the influx of granulocytes and macrophages. A certain induction of local bladder cellularity was also noted in mice which received s.c. injections of PstS1 but no intravesical instillation of the antigen (group 3). The reason for this effect of s.c. PstS1 injection on bladder cellularity is unclear.

## Discussion

BCG therapy is a clinically successful therapy in the treatment of bladder cancer but its acceptance is hampered by hazards and side effects related to the use of viable mycobacteria. The aim of this study was to evaluate the anti-tumor potential of the well-defined, non-viable mycobacterial antigen PstS1.

To this end we first tested the potential of recombinant PstS1 to activate human PBMC cultures. In this series of experiments PstS1 induced strong cytotoxicity, proliferation and IFN-γ production in human PBMC. Ten μg/ml PstS1 were found to be optimal for activation of human PBMC. We used BCG lyophilisate as a reference stimulus in these experiments as we have previously described BCG as a potent activator of human PBMC functions [27]. PstS1 followed a similar time kinetic as BCG in the stimulation of human PBMC, albeit activation of PBMC functions was somewhat lower with PstS1 compared with BCG. PstS1 was previously shown to induce profound cellular immunity in different vaccination studies [28-30]. Therefore, our results further confirm the potential of PstS1 to function as a potent inducer of T-helper 1 and cell-mediated immune responses.

While IFN-γ is a key cytokine in TH1 immune responses produced by activated lymphocytes, IL-12 is mainly produced by monocytes/macrophages as well as dendritic cells and important in the early phase of cellular immunity [31]. IL-12 is a potent inducer of anti-tumor immunity [32] and acts together with IFN-γ and TNF-α in a positive feedback loop [25,33,34]. Although BCG mycobacteria are relatively weak inducers of bioactive IL-12p70 in human dendritic cells in the absence of additional costimulation like CD154 or IFN-γ (figure 3b), IL-12 has been shown to be essential for therapeutic efficacy in experimental BCG immunotherapy of bladder cancer [24]. Given the importance of dendritic cells for the initiation of anti-tumor immune responses [35] and as a source of IL-12 and TNF-α we tested the capacity of recombinant PstS1 to activate human monocyte-derived dendritic cells. Our results clearly identified PstS1 as a potent stimulator of human DCs as it provoked upregulation of CD83 and CD86 surface expression as well as induction of cytokines. Of interest is the expression of high amounts of bioactive IL-12 after stimulation of DCs with PstS1 as only small amounts of this cytokine are produced after challenge with BCG or other mycobacterial antigens (figure 3 and unpublished observations).

The encouraging *in vitro *experiments then prompted us to evaluate the immunotherapeutic potential of PstS1 in a well-established murine model of experimental bladder cancer [20]. The effect of prior sensitization or responsiveness to mycobacterial antigens for the effectiveness and outcome of subsequent immunotherapy with BCG is still a matter of intensive debate [21-23]. Taking this into account we took advantage of the fact that we had in hand a well-defined, recombinant antigen already evaluated in vaccination studies against mycobacterial infections and included a prime-boost treatment regimen into our experimental immunotherapy protocol. The main objective of our series of *in vivo *experiments was to determine the anti-tumor potential of local instillations of recombinant PstS1 and to compare the effect of intravesical PstS1 in sensitized and non-sensitized mice. Because adsorption of PstS1 to L-particles has previously been described as very efficient in inducing specific cellular immunity to this antigen [29], we adopted this method of sensitization for our treatment protocol. Using a protocol of four weekly instillations of PstS1 into the bladder (adopted from the treatment schedule in use for immunotherapy with BCG) we observed a strong therapeutic effect of PstS1 instillations. Intravesical PstS1 significantly prolonged survival of mice and induced systemic immune responses and cellular infiltration of the bladder with different subpopulations of leukocytes. The anti-tumor effect of PstS1 was already evident after intravesical instillation of PstS1 only but was further enhanced after s.c. injection of empty L-particles (Fig. 5). Unexpectedly, sensitization of mice with PstS1-loaded particles almost completely abrogated the therapeutic effect of intravesical PstS1 (Fig. 4). In order to test whether the negative effect of prior sensitization was specific for PstS1 or could also be induced by s.c. injection of L-particles loaded with an irrelevant antigen, we compared the therapeutic effect of intravesical PstS1 combined with a) s.c. sensitization with BSA-loaded L-particles, b) s.c. injection of empty L-particles and c) s.c. injection of PBS only (Fig. 5). Clearly, the injection of BSA-loaded particles did not influence the therapeutic effect of intravesical PstS1 indicating that only PstS1-specific previous sensitization is detrimental (Fig. 5a and 5c). We analyzed the systemic serum antibody response, activation of splenocytes and the local cellular infiltration of the bladder wall in sensitized and non-sensitized mice (Fig. 6) in order to understand and explain the remarkable therapeutic potential of intravesical PstS1 as well as the negative effect of prior specific sensitization. Interestingly, even without sensitization, instillation of PstS1 into the bladder provoked a systemic anti-PstS1 response visualized by murine anti-PstS1 serum antibodies and splenocyte proliferation after rechallenge *in vitro*. In addition to this systemic immune activation, intravesical PstS1 also induced the local influx of lymphocytes, macrophages and granulocytes into the bladder. While only a minimal influx of CD8-cells could be observed after instillation of PstS1, a considerable infiltration of the bladder with granulocytes, macrophages and CD4-cells was noted. Although prior specific sensitization with PstS1 completely abrogated the anti-tumor effect of intravesical PstS1, the cellular infiltration of the bladder wall remained essentially unchanged when comparing sensitized and non-sensitized animals. In the splenocyte restimulation assay an enhanced response of two out of four mice was noted in the group of mice which received s.c. PstS1 followed by intravesical PstS1 compared with the two groups which received either treatment alone (Fig. 6). This indicates that the combination of s.c. sensitization and intravesical treatment indeed augmented the systemic immune response at least in some animals. Surprisingly, this enhanced systemic immune response coincided with an abrogation of tumor-therapeutic efficacy. A possible explanation for this phenomenon could be that the anti-PstS1 antibodies, which were induced after s.c. priming would bind to the recombinant PstS1 shortly after instillation into the bladder and thereby neutralize its function and anti-tumor effect. However, as mentioned earlier, even in the PstS1-specific prime-boost treatment regimen an unchanged cellular infiltration of the bladder wall was noted. Alternatively, the sensitization might render the subsequent local immunotherapeutic immune response insufficient, because the host immune system is still actively responding to the priming at the onset of immunotherapy. This situation might be called "immune exhaustion" making the host unable to mount a sufficient local anti-tumor immune response while still responding to the specific priming. Further experiments are needed to clarify whether a modification of the time schedule of such a "prime-immunotherapy" protocol could prevent the negative effects observed in this study and might even enhance immunotherapy with PstS1.

Nonetheless, our combined *in vitro *and *in vivo *analyses clearly identified PstS1 from *M. tuberculosis *as a potent immunostimulant and a potential immunotherapeutic anti-cancer agent for topical treatment strategies. Our data do not show and do not imply that PstS1 is the major or only immunostimulatory component of whole BCG mycobacteria in BCG immunotherapy of bladder cancer.

## Conclusions

We have identified the immunodominant mycobacterial PstS1 antigen as a potent biological response modifier for tumor immunotherapy. Using a human *in vitro *system of PBMC activation and a murine model of experimental bladder cancer immunotherapy we could show strong immunostimulatory capacity of PstS1 as well as significant anti-tumor activity. In a model of prime-boost immunotherapy we observed that antigen-specific sensitization might jeopardize the positive effects of topical immunotherapy and therefore has to be considered and evaluated with caution. GMP-production of PstS1 and a clinical trial in humans is currently being established and might open the door for an efficient and safe alternative in the field of bladder cancer immunotherapy.

## Competing interests

The author(s) declare that they have no competing interests.

## Authors' contributions

CS carried out most of the experiments, provided experimental protocols and prepared the initial version of the manuscript. ABu contributed to figure 2 and worked on the manuscript. GB performed the immunohistochemistry and cell culture. RS and FJ performed the α-PstS1 IgG ELISA and provided the recombinant PstS1. MS, ABö and SB jointly participated in fund raising and coordination. SB is the principal investigator, edited the manuscript and advised CS on experimental design and study concept.

## Pre-publication history

The pre-publication history for this paper can be accessed here:



## Figures and Tables

**Table 1:** Serum antibody response after PstS1 immunotherapy

**S.C. / Intravesical Treatment**	**Responders in %**
PBS/PBS	**0**
L-Particle/PBS	**0**
L-Particle-PstS1/PBS	**100**
PBS/PstS1	**100**
L-particle/PstS1	**100**
L-particle-PstS1/PstS1	**100**

Five mice per group were sensitized s.c. and treated intravesically on days 10, 17, 24 and 31 as indicated in the table. On day 32 serum of mice was obtained and analyzed for anti-PstS1 IgG by ELISA. The percentage of mice with positive anti-PstS1 antibody responses is indicated.

**Table 2:** Local immune response in the bladder wall after PstS1 immunotherapy

**group**	**S.C. / Intravesical Treatment**	**CD11b**	**GR-1**	**CD4**	**CD8**	**CD11c**
1	PBS/PBS	11± 8	3 ± 2	28 ± 24	8 ± 9	2,2 ± 1,7
2	L-Particle/PBS	6 ± 7	3 ± 2	17 ± 6	4 ± 3	1,6 ± 1,4
3	L-Particle-PstS1/PBS	24 ± 20	4 ± 7	69 ± 60	16 ± 15	22,2 ± 24,4
4	PBS/PstS1	86 ± 46	44 ± 30	45 ± 18	10 ± 4	1,9 ± 2,1
5	L-Particle/PstS1	114 ± 25	56 ± 20	56 ± 16	13 ± 14	3 ± 2,4
6	L-Particle-PstS1/PstS1	143 ± 84	49 ± 15	75 ± 29	36 ± 29	4,5 ± 4,2

Five mice per group were sensitized s.c. and treated intravesically on days 10, 17, 24 and 31 as indicated in the table. On day 32 bladders were dissected and used for histochemistry. CD11b: Granulocytes and Macrophages, GR-1: Granulocytes, CD4: CD4-Lymphocytes, CD8: CD8-Lymphocytes, CD 11c: Dendritic cells. The number of positive cells in the entire bladder specimens was counted using visual fields and light microscopy. The data are depicted as the mean number of positive cells per visual field ± standard deviation (n = five mice per group).

## References

[B1] Lamm DL, Blumenstein BA, Crawford ED, Montie JE, Scardino P, Grossman HB, Stanisic TH, Smith JAJ, Sullivan J, Sarosdy MF, . (1991). A randomized trial of intravesical doxorubicin and immunotherapy with bacille Calmette-Guerin for transitional-cell carcinoma of the bladder. N Engl J Med.

[B2] Herr HW, Schwalb DM, Zhang ZF, Sogani PC, Fair WR, Whitmore WFJ, Oettgen HF (1995). Intravesical bacillus Calmette-Guerin therapy prevents tumor progression and death from superficial bladder cancer: ten-year follow-up of a prospective randomized trial. J Clin Oncol.

[B3] Malmstrom PU, Wijkstrom H, Lundholm C, Wester K, Busch C, Norlen BJ (1999). 5-year followup of a randomized prospective study comparing mitomycin C and bacillus Calmette-Guerin in patients with superficial bladder carcinoma. Swedish-Norwegian Bladder Cancer Study Group. J Urol.

[B4] Herr HW, Badalament RA, Amato DA, Laudone VP, Fair WR, Whitmore WFJ (1989). Superficial bladder cancer treated with bacillus Calmette-Guerin: a multivariate analysis of factors affecting tumor progression. J Urol.

[B5] Cymes M, Fleischmann JD, Smith E (1992). Invasive bladder cancer treated with intravesical BCG. J Urol.

[B6] Lamm DL (1992). Complications of bacillus Calmette-Guerin immunotherapy. Urol Clin North Am.

[B7] Steg A, Adjiman S, Debre B (1992). BCG therapy in superficial bladder tumours--complications and precautions. Eur Urol.

[B8] Armstrong RW (1991). Complications after intravesical instillation of bacillus Calmette-Guerin: rhabdomyolysis and metastatic infection. J Urol.

[B9] Zlotta AR, Van Vooren JP, Denis O, Drowart A, Daffe M, Lefevre P, Schandene L, De Cock M, De Bruyn J, Vandenbussche P, Jurion F, Palfliet K, Simon J, Schulman CC, Content J, Huygen K (2000). What are the immunologically active components of bacille Calmette-Guerin in therapy of superficial bladder cancer?. Int J Cancer.

[B10] Rohde D, Gastl G, Biesterfeld S, Plante M, Jakse G (1997). Local expression of cytokines in rat bladder carcinoma tissue after intravesical treatment with Nocardia rubra cell wall skeleton and bacille-Calmette-Guerin. Urol Res.

[B11] de Boer EC, De Reijke TM, Vos PC, Kurth KH, Schamhart DH (2000). Immunostimulation in the urinary bladder by local application of Nocardia rubra cell-wall skeletons (Rubratin) and bacillus Calmette-Guerin as therapy for superficial bladder cancer: a comparative study. Clin Infect Dis.

[B12] Morales A, Chin JL, Ramsey EW (2001). Mycobacterial cell wall extract for treatment of carcinoma in situ of the bladder. J Urol.

[B13] Vordermeier HM, Coombes AG, Jenkins P, McGee JP, O'Hagan DT, Davis SS, Singh M (1995). Synthetic delivery system for tuberculosis vaccines: immunological evaluation of the M. tuberculosis 38 kDa protein entrapped in biodegradable PLG microparticles. Vaccine.

[B14] Andersen AB, Hansen EB (1989). Structure and mapping of antigenic domains of protein antigen b, a 38,000-molecular-weight protein of Mycobacterium tuberculosis. Infect Immun.

[B15] Braibant M, Lefevre P, de Wit L, Peirs P, Ooms J, Huygen K, Andersen AB, Content J (1996). A Mycobacterium tuberculosis gene cluster encoding proteins of a phosphate transporter homologous to the Escherichia coli Pst system. Gene.

[B16] Espitia C, Mancilla R (1989). Identification, isolation and partial characterization of Mycobacterium tuberculosis glycoprotein antigens. Clin Exp Immunol.

[B17] Young DB, Garbe TR (1991). Lipoprotein antigens of Mycobacterium tuberculosis. Res Microbiol.

[B18] Ivanyi J (1994). Tuberculosis: Pathogenesis, Protection and Control. Bloom B (ed) ASMp Press: Washington.

[B19] Singh M, Andersen AB, McCarthy JE, Rohde M, Schutte H, Sanders E, Timmis KN (1992). The Mycobacterium tuberculosis 38-kDa antigen: overproduction in Escherichia coli, purification and characterization. Gene.

[B20] Gunther JH, Jurczok A, Wulf T, Brandau S, Deinert I, Jocham D, Bohle A (1999). Optimizing syngeneic orthotopic murine bladder cancer (MB49). Cancer Res.

[B21] Shinka T, Hirano A, Uekado Y, Ohkawa T (1990). Clinical study of prognostic factors of superficial bladder cancer treated with intravesical bacillus Calmette-Guerin. Br J Urol.

[B22] Saint F, Salomon L, Quintela R, Cicco A, Hoznek A, Abbou CC, Chopin DK (2003). Do prognostic parameters of remission versus relapse after Bacillus Calmette-Guerin (BCG) immunotherapy exist?. analysis of a quarter century of literature. Eur Urol.

[B23] Bilen CY, Inci K, Erkan I, Ozen H (2003). The predictive value of purified protein derivative results on complications and prognosis in patients with bladder cancer treated with bacillus Calmette-Guerin. J Urol.

[B24] Riemensberger J, Bohle A, Brandau S (2002). IFN-gamma and IL-12 but not IL-10 are required for local tumour surveillance in a syngeneic model of orthotopic bladder cancer. Clin Exp Immunol.

[B25] O'Donnell MA, Luo Y, Chen X, Szilvasi A, Hunter SE, Clinton SK (1999). Role of IL-12 in the induction and potentiation of IFN-gamma in response to bacillus Calmette-Guerin. J Immunol.

[B26] Suttmann H, Jacobsen M, Reiss K, Jocham D, Bohle A, Brandau S (2004). Mechanisms of bacillus Calmette-Guerin mediated natural killer cell activation. J Urol.

[B27] Brandau S, Bohle A, Thanhauser A, Ernst M, Mattern T, Ulmer AJ, Flad HD (2000). In vitro generation of bacillus Calmette-Guerin-activated killer cells. Clin Infect Dis.

[B28] Vordermeier HM, Harris DP, Mehrotra PK, Roman E, Elsaghier A, Moreno C, Ivanyi J (1992). M. tuberculosis-complex specific T-cell stimulation and DTH reactions induced with a peptide from the 38-kDa protein. Scand J Immunol.

[B29] Venkataprasad N, Coombes AG, Singh M, Rohde M, Wilkinson K, Hudecz F, Davis SS, Vordermeier HM (1999). Induction of cellular immunity to a mycobacterial antigen adsorbed on lamellar particles of lactide polymers. Vaccine.

[B30] Sfondrini L, Rodolfo M, Singh M, Colombo MP, Colnaghi MI, Menard S, Balsari A (2000). Cooperative effects of Mycobacterium tuberculosis Ag38 gene transduction and interleukin 12 in vaccination against spontaneous tumor development in proto-neu transgenic mice. Cancer Res.

[B31] Macatonia SE, Hosken NA, Litton M, Vieira P, Hsieh CS, Culpepper JA, Wysocka M, Trinchieri G, Murphy KM, O'Garra A (1995). Dendritic cells produce IL-12 and direct the development of Th1 cells from naive CD4+ T cells. J Immunol.

[B32] Shurin MR, Esche C, Peron JM, Lotze MT (1997). Antitumor activities of IL-12 and mechanisms of action. Chem Immunol.

[B33] Flesch IE, Hess JH, Huang S, Aguet M, Rothe J, Bluethmann H, Kaufmann SH (1995). Early interleukin 12 production by macrophages in response to mycobacterial infection depends on interferon gamma and tumor necrosis factor alpha. J Exp Med.

[B34] Tripp CS, Wolf SF, Unanue ER (1993). Interleukin 12 and tumor necrosis factor alpha are costimulators of interferon gamma production by natural killer cells in severe combined immunodeficiency mice with listeriosis, and interleukin 10 is a physiologic antagonist. Proc Natl Acad Sci U S A.

[B35] Shurin MR (1996). Dendritic cells presenting tumor antigen. Cancer Immunol Immunother.

